# Occult Intertrochanteric Fracture Detected by Bone Scan Imaging: A Case Report

**DOI:** 10.7759/cureus.64815

**Published:** 2024-07-18

**Authors:** Ahmad N Boeisa, Hassan A Alghanim, Abdualziz Almutlaq, Mohammed Al-Saeed, Munirah Alshaikhmubarak

**Affiliations:** 1 Pediatric Orthopedics, Almoosa Specialist Hospital, Al Mubarraz, SAU; 2 Surgery, King Faisal University, Dammam, SAU; 3 Medicine, King Faisal University, Dammam, SAU; 4 Orthopedics and Traumatology, King Fahad Hospital, Riyadh, SAU

**Keywords:** orthopedic trauma, hip fracture diagnosis, orthopedic surgery, hidden intertrochanteric fracture, occult intertrochanteric fracture

## Abstract

Femoral intertrochanteric fractures can be occult and not visible on plain radiographs, even when there is a high clinical suspicion. This case study reports an occult intertrochanteric fracture that was diagnosed using a bone scan rather than an MRI or CT scan.

A 91-year-old woman arrived at the emergency room with a complaint of left hip pain after slipping at home. Clinical examination revealed tenderness, mild swelling, limited range of motion, and an inability to bear weight. Radiographs and CT scans of the hip and pelvis showed no evidence of a fracture. An MRI was planned, but the patient's agitation resulted in improper images. Consequently, a bone scan identified an ill-defined focal area with slightly increased activity, consistent with an intertrochanteric femur fracture. In such challenging scenarios, bone scans can still serve as an alternative diagnostic tool, aiding in clinical decision-making.

## Introduction

Hip fractures predominantly affect female and elderly individuals in their sixth decade of life and are considered a global health challenge due to their status as a leading cause of disability and their noteworthy one-year mortality rate of up to 30%. The estimated prevalence of all hip fractures, including occult hip fractures, reaches up to 10% [1-3]. An occult intertrochanteric fracture occurs between the greater and lesser trochanters that is not detectable through standard radiographic evaluation until days after the injury. The main clinical signs of intertrochanteric fractures include ecchymosis, hip pain, and swelling. Typically, distal pulses and nerve function remain normal. Accurate and early diagnosis of hip fractures is crucial due to their association with a higher risk of morbidity and mortality [4]. Plain radiography is the initial imaging modality of choice in the emergency department. However, due to the fact that an occult fracture shows negative results on a plain radiograph, an accurate diagnosis might be challenging [5]. Studies indicate that MRI is regarded as the gold standard for certain conditions, while CT is often the first-line investigative tool due to its high sensitivity, specificity, and accessibility. Despite these advantages of CT, MRI is superior for detecting fractures because it provides more detailed images of soft tissues and bone marrow, which can reveal subtle fractures that CT might miss. Alternatively, bone scans are considered an alternative option to diagnose occult hip fractures, offering advantages such as examination of the entire body, cost-effectiveness, and high sensitivity [6]. This case study reports an occult intertrochanteric fracture that was diagnosed using a bone scan rather than an MRI or CT scan.

## Case presentation

**Figure 1:**
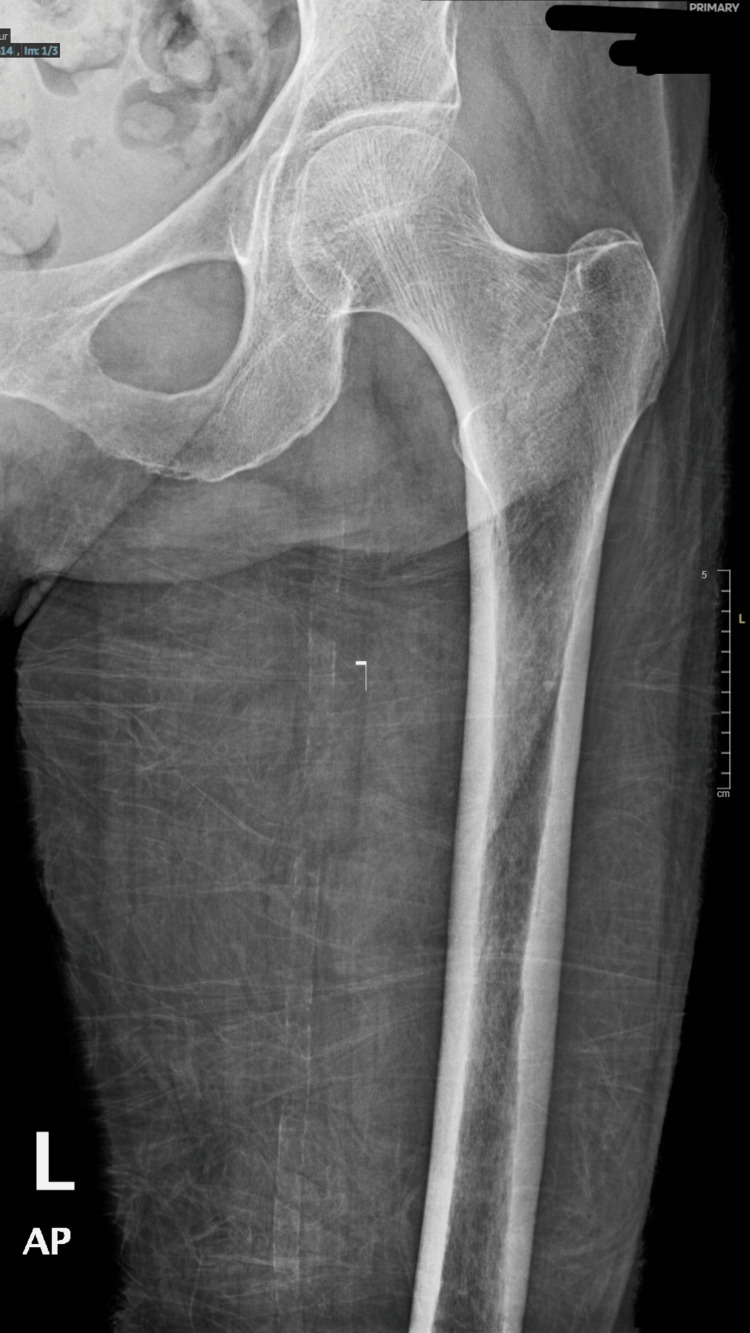
Plain radiograph shows no definite fracture lines or osseous lesions

A 91-year-old Saudi woman was presented to the emergency department complaining of left hip and pelvic pain after slipping down at home. She had a history of diabetes mellitus, hypertension, dyslipidemia, and osteoporosis. On examination, she was conscious and oriented, and the Glasgow Coma Scale was 15/15. The patient experienced tenderness and mild swelling over the left hip joint, a limited range of motion, and an inability to walk. The patient was then referred to orthopedics due to the suspicion of a left femoral neck fracture. In the context of clinical investigation, plain radiographs yielded negative results (Figure 1).

**Figure 2:**
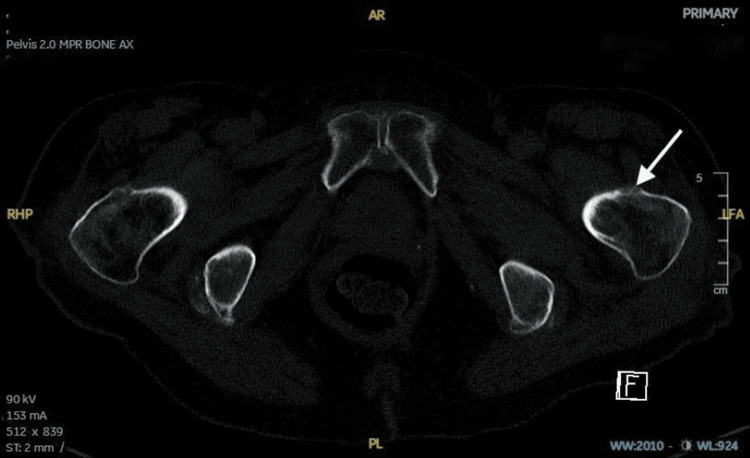
CT scan of the pelvis without contrast. Axial image: suspected hazy cortical outline with suspected cortical interruption along the anterior aspect of the left femoral neck CT: computed tomography

**Figure 3:**
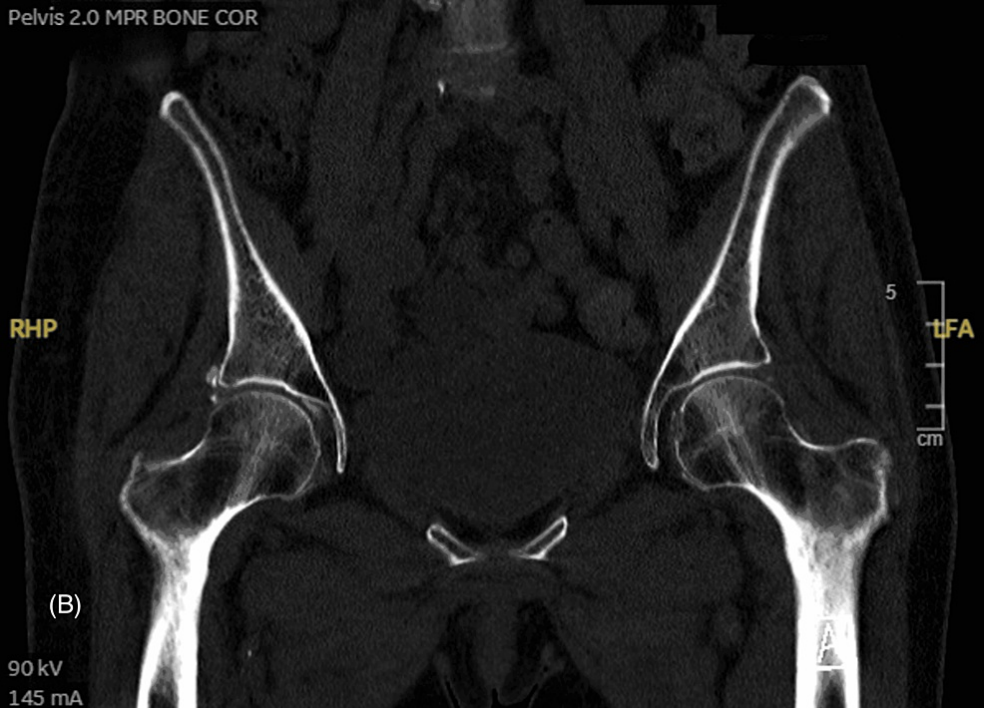
CT scan of the pelvis without contrast coronal image CT: computed tomography

**Figure 4:**
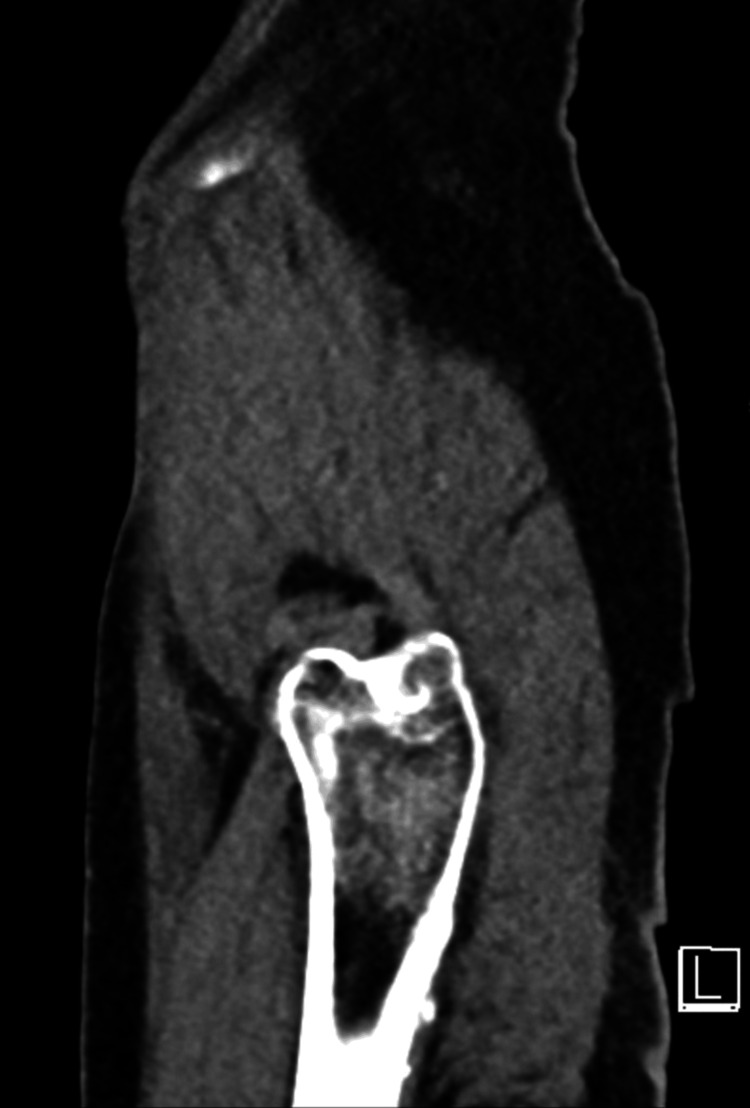
Sagittal image did not show any established displaced fracture lines along the visualized bones

**Figure 5:**
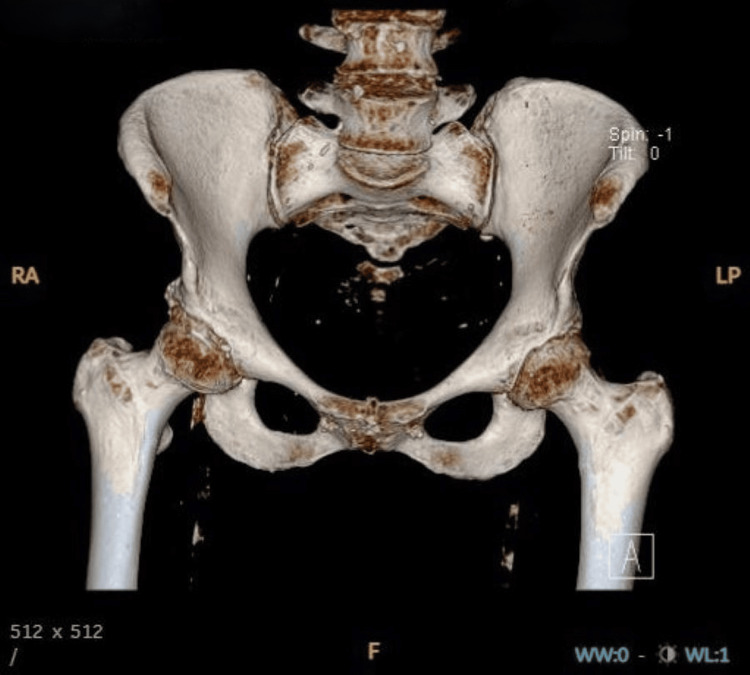
3D CT scan of the pelvis without contrast, anteroposterior view image 3D: three dimensional, CT: computed tomography

However, due to high clinical suspicion, further imaging was done using a CT scan, which revealed a discernible hazy cortical outline, specifically noting a suspected cortical interruption along the anterior aspect of the left femoral neck but no clear evidence of a displaced fracture line. This manifestation was most evident in the axially reformatted images, and no significant displacement or other established displaced fracture lines were identified across the visualized skeletal structures (Figures 2-5).

**Figure 6:**
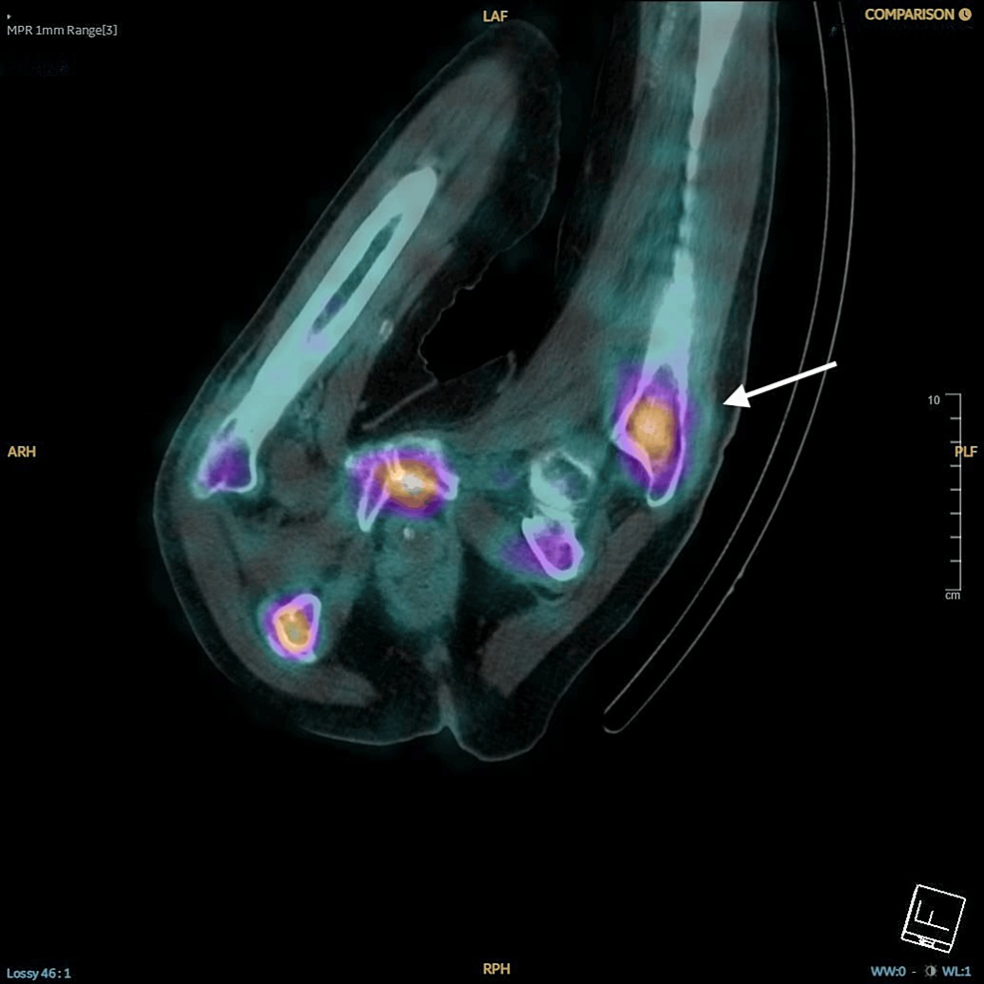
Bone scan image shows an ill-defined focal area of mildly increased tracer uptake at the intertrochanteric/subtrochanteric level of the left femur. There is no evidence of a clear fracture line, indicating the presence of an occult intertrochanteric fracture

Then, an MRI was planned, but the patient was unable to comply with the instructions for the MRI session for an extended period, resulting in the acquisition of improper images because of the patient’s agitation. Consequently, the patient was admitted to address pain management and determine the final course of action. A bone scan of the left hip was performed after the injection of 20 mCi 99mTc-MDP, which revealed an ill-defined focal area with mildly increased activity. The observed pattern was indicative of a fracture situated at the intertrochanteric level of the femur (Figure 6).

**Figure 7:**
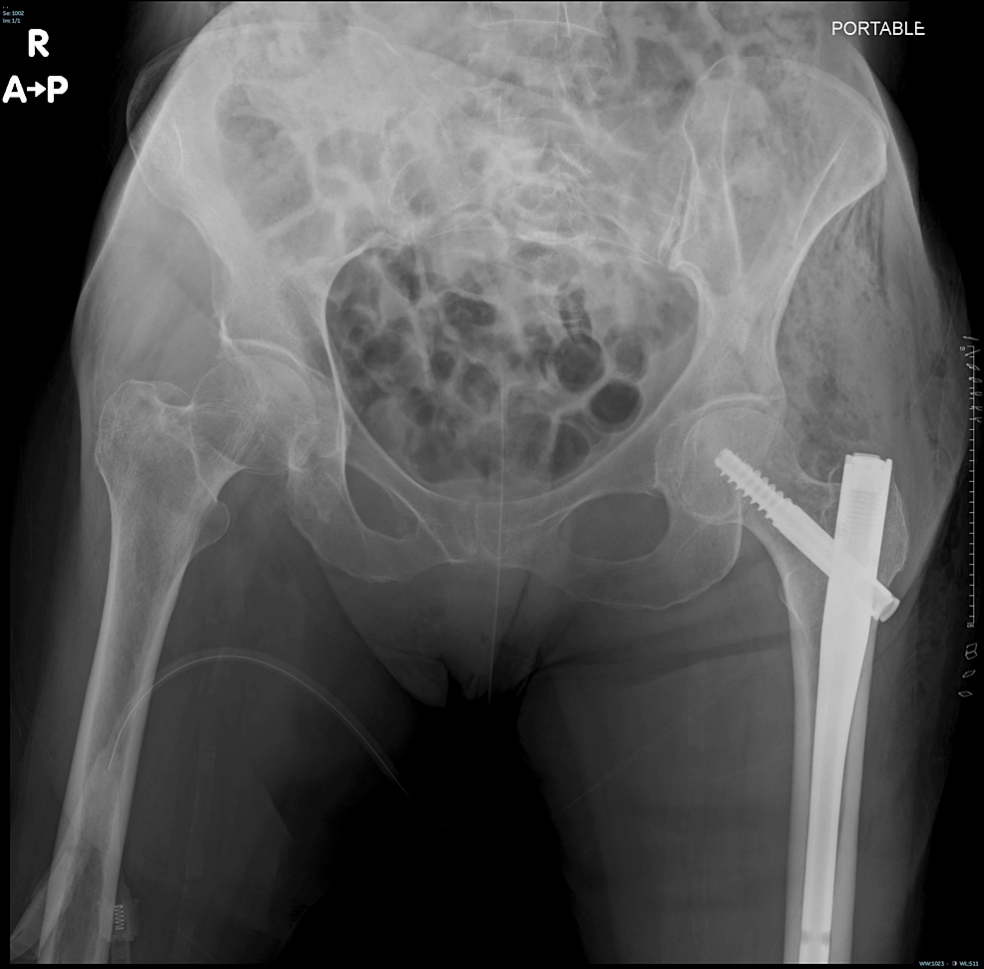
Plain radiograph taken immediately after surgery shows internal fixation of the left femoral neck with an intramedullary nail and screw. No other definite recent fracture lines are seen

The surgical setting involved the patient's supine positioning on a traction table under spinal anesthesia. The procedural steps encompassed a proximal incision to the greater trochanter, the insertion of a guide wire into the femur, proximal reaming, and the subsequent insertion of a 10 x 200 mm Gamma nail at a 125-degree angle. A guide to the neck was concurrently introduced, followed by reaming of the neck and the insertion of an 800-mm-long lag screw (Figure 7). The anticipated outcome of the surgical intervention is directed toward achieving stability, fostering proper healing, and mitigating the patient's pain. The overarching goal is to mitigate the patient's left hip pain and reinstate mobility. Tables 1-2 show the details of the medications administered pre- and post-operation, respectively.

**Table 1:** Medications administered pre-operative STAT: immediately, PRN: as needed, TID: three times a day, BID: twice a day, DVT: deep venous thrombosis

Medication	Dosage	Route	Frequency	Indication	Period
Paracetamol	1000 mg	IV drip	STAT	Pain/fever	-
Morphine	3 mg	IV push	STAT	Pain	-
Insulin aspart	Sliding scale	Subcutaneous	Multiple times a day	Blood glucose control	-
Insulin glargine	16 units	Subcutaneous	Ongoing	Blood glucose control	Ongoing
Metformin	500 mg	Oral	-	Diabetes management	Pre-operation
Vildagliptin	50 mg	Oral	-	Diabetes management	Pre-operation
Rosuvastatin	10 mg	Oral	Once daily	Lipid management	Pre-operation
Telmisartan	80 mg	Oral	Once daily	Hypertension management	Pre-operation
Ondansetron	8 mg	IV push	PRN	Nausea or vomiting	Pre-operation
Tramadol	100 mg	IV drip	TID PRN	Pain	Pre-operation
Lornoxicam	8 mg	IV drip	BID	Pain/inflammation	Pre-operation
Enoxaparin	40 mg	Subcutaneous	Once daily	DVT prophylaxis	Pre-operation
Calcium carbonate with vitamin D3, magnesium, and zinc	-	Oral	Once daily	Fracture management	Pre-operation

**Table 2:** Medications administered post-operation IV: intravenous, TID: three times a day, BID: twice a day, PRN: as needed, DVT: deep vein thrombosis

Medication	Dosage	Route	Frequency	Indication	Period
Paracetamol	1000 mg	IV drip	TID	Pain/fever	Post-operation
Rosuvastatin	10 mg	Oral	Once daily	Lipid management	Post-operation
Ondansetron	8 mg	IV push	BID PRN	Nausea or vomiting	Post-operation
Tramadol	100 mg	IV drip	TID PRN	Pain	Post-operation
Lornoxicam	8 mg	IV drip	BID	Pain/Inflammation	Post-operation
Telmisartan	80 mg	Oral	Once daily	Hypertension management	Post-operation
Enoxaparin	40 mg	Subcutaneous	Once daily	DVT prophylaxis	Post-operation
Calcium carbonate with vitamin D3, magnesium, and zinc	-	Oral	Once daily	Fracture management	Post-operation

In the immediate post-operative clinical evaluation, three parameters were assessed, including pain, neurovascular status, and immobilization, and were found to be within normal ranges. Pain management measures have been effective in addressing discomfort, and the patient's sensory and motor functions and vascular status were within expected parameters. Immobilization techniques have been appropriately applied to maintain joint stability, contributing to the initial success of the surgical intervention. The patient's hemoglobin levels decreased within the normal range, from 11.1 to 10.3 grams per deciliter (g/dL) (Table 3).

**Table 3:** Patient's relevant lab reports Hb: hemoglobin, HCT: hematocrit, WBC: white blood cell, RBC: red blood cell, MCH: mean corpuscular hemoglobin

Test	Pre-operation	Post-operation	Reference range
Hb	11.10	10.30	13.5-17.5 g/dL (male)/12.0-15.5 g/dL (female)
HCT	34.80	31.90	38.3-48.6% (male)/35.5-44.9% (female)
WBC count	7.39	7.32	4.0-11.0 x 10^9/L
RBC count	4.00	3.71	4.7-6.1 x 10^12/L (male)/4.2-5.4 x 10^12/L (female)
MCH	27.70	27.80	27.5-33.2 pg

Afterward, the patient started a gradual rehabilitation program. A three-week post-operative assessment revealed normal findings on the X-ray. Subsequently, at the four-week mark, the patient commenced a home-based physiotherapy regimen. The rehabilitative program was designed to optimize the patient's hip range of motion and contribute to a successful recovery process.

**Figure 8:**
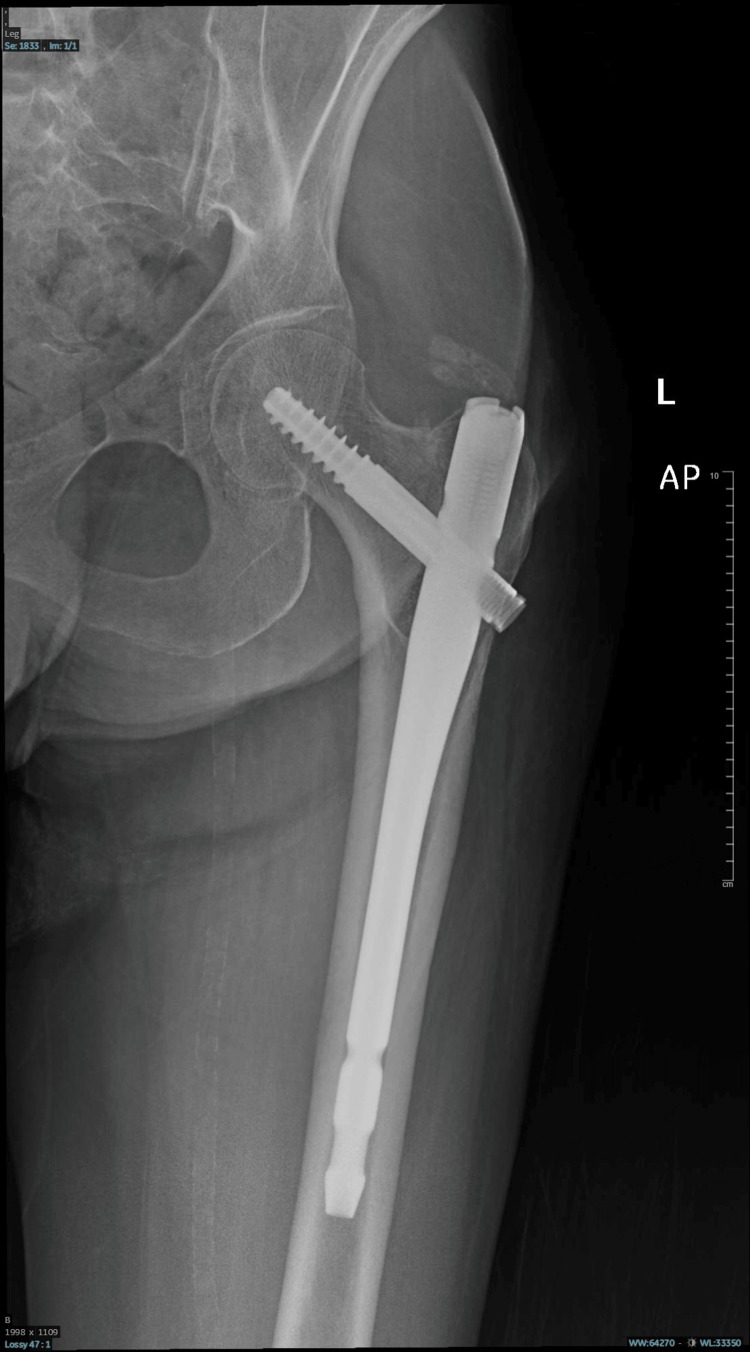
Diffuse decreased bone density with internal fixation of the left femoral neck using an intra-medullary nail and screw. No other definite recent fracture line was seen

To further quantify the clinical outcomes, we performed the Harris Hip Score at the six-week follow-up, which showed significant improvement in pain and functional status, with a score of 85 out of 100, indicating good hip function. Additionally, the Visual Analog Scale for pain decreased from 8/10 pre-operatively to 2/10 post-operatively. Furthermore, a follow-up X-ray was performed one month and two weeks post-operation, as shown in Figure 8.

## Discussion

History and physical examination are essential for raising suspicion of an occult intertrochanteric femoral fracture [7]. During the examination, there may be instability when bearing weight, pain during range of motion, pain during straight leg raises, and limitations in hip range of motion. Additionally, pain upon palpation and a lack of improvement in pain over time may be observed [8-10]. In cases where there is a clinical suspicion of a hip fracture, the initial imaging usually involves a plain radiograph, which has the ability to detect the majority of hip fractures. However, the detection of occult fractures on plain radiographs is impeded by the presence of a high percentage of trabecular bone in the proximal femur, which makes the detection of disruptions more challenging compared to cortical bone [11]. Therefore, the growing frequency of clinically significant hip fractures not detected on plain radiographs emphasizes the need for advanced imaging such as MRI and CT. MRI is considered the gold-standard method, with nearly 100% sensitivity and specificity compared to other modalities. Additionally, MRI can prevent associated risks and mortality. However, it may not be as easily accessible, cost-effective, or prompt as a CT scan [6,7]. Recent studies have shown that CT is being used as a first-line imaging modality due to its high sensitivity, specificity, easy availability, low cost, and fast scan time. However, it can yield false-negative results when compared to MRI, particularly within the initial 24 hours [7]. Bone scintigraphy can serve as an alternative diagnostic tool for identifying occult intertrochanteric fractures, in particular when CT or MRI are contraindicated or not feasible due to the inability to comply with the instructions for a long time, often due to agitation in the elderly. This imaging test offers advantages such as sensitivity (93%) and specificity (95%) in the detection of occult intertrochanteric fractures [12,13]. However, it is important to keep in mind that using bone scintigraphy may erroneously identify pathological conditions such as contusion, synovitis, or degenerative arthritis in elderly patients as occult intertrochanteric fractures. Also, the specificity of a bone scan depends heavily on the clinical presentation and history since increased radiotracer uptake is associated with various pathological conditions, including fractures, infections, malignancies, and, less frequently, bone-related disorders like Paget's disease and fibrous dysplasia [14].

## Conclusions

MRI is the gold-standard method due to its high sensitivity and specificity, nearly 100%; however, CT scans are considered the first-line investigative modality due to their accessibility and high accuracy. This case has shown that the CT scan yielded negative results, and the MRI was not feasible due to the patient's inability to comply with the instructions for an extended period of time. In such challenging scenarios, bone scans can still be used as an alternative diagnostic tool and could facilitate clinical decisions.
